# Systematic Comparison of Two Animal-to-Human Transmitted Human Coronaviruses: SARS-CoV-2 and SARS-CoV

**DOI:** 10.3390/v12020244

**Published:** 2020-02-22

**Authors:** Jiabao Xu, Shizhe Zhao, Tieshan Teng, Abualgasim Elgaili Abdalla, Wan Zhu, Longxiang Xie, Yunlong Wang, Xiangqian Guo

**Affiliations:** 1 Department of Preventive Medicine, Institute of Biomedical Informatics, Bioinformatics Center, Henan Provincial Engineering Center for Tumor Molecular Medicine, School of Basic Medical Sciences, Henan University, Kaifeng 475004, China; xujiabao0907@126.com (J.X.); zhaoshizhe1126@126.com (S.Z.); xiaoshan1220@163.com (T.T.); 2 Department of Clinical Laboratory Sciences, College of Applied Medical Sciences, Jouf University, Sakaka 2014, Saudi Arabia; gasimmicro@gmail.com; 3 Department of Anesthesia, Stanford University, Stanford, CA 94305, USA; ms.wanzhu@gmail.com; 4 Henan Bioengineering Research Center, Zhengzhou 450046, China

**Keywords:** coronaviruses, SARS-CoV-2, SARS-CoV, genomic comparison, proteomic comparison, pathogenic mechanism, clinical manifestations

## Abstract

After the outbreak of the severe acute respiratory syndrome (SARS) in the world in 2003, human coronaviruses (HCoVs) have been reported as pathogens that cause severe symptoms in respiratory tract infections. Recently, a new emerged HCoV isolated from the respiratory epithelium of unexplained pneumonia patients in the Wuhan seafood market caused a major disease outbreak and has been named the severe acute respiratory syndrome coronavirus 2 (SARS-CoV-2). This virus causes acute lung symptoms, leading to a condition that has been named as “coronavirus disease 2019” (COVID-19). The emergence of SARS-CoV-2 and of SARS-CoV caused widespread fear and concern and has threatened global health security. There are some similarities and differences in the epidemiology and clinical features between these two viruses and diseases that are caused by these viruses. The goal of this work is to systematically review and compare between SARS-CoV and SARS-CoV-2 in the context of their virus incubation, originations, diagnosis and treatment methods, genomic and proteomic sequences, and pathogenic mechanisms.

## 1. Introduction

Coronaviruses (CoVs) are a group of viruses that co-infect humans and other vertebrate animals. CoV infections affect the respiratory, gastrointestinal, liver, and central nervous systems of humans, livestock, birds, bats, mice, and many other wild animals [1,2,3]. For example, severe acute respiratory syndrome (SARS) in 2002 and the Middle East respiratory syndrome (MERS) in 2012 were both coronaviruses that transmitted from animals to humans [4,5]. The source of unexplained pneumonia was first discovered in Wuhan in Dec, 2019, and SARS-CoV-2, a new coronavirus, was isolated from the respiratory epithelium of patients. It belongs to a new evolutionary branch within the CoV. On Feb. 11th, 2020, the new coronavirus was officially renamed “SARS-CoV-2” from “2019-nCoV” [6]. The disease caused by SARS-CoV-2 was called “coronavirus disease 2019” (COVID-19) [7]. According to the data released by the National Health Commission of the People’s Republic of China, SARS-CoV-2 was most likely transmitted from wild bats to humans, and all the above three CoVs can transmit from person to person [8,9,10]. SARS-CoV-2 shares a highly similar gene sequence and behavior pattern with SARS-CoV [11]. This paper summarized the similarities and differences between SARS-CoV-2 and SARS-CoV, both of which cause major disease outbreaks in China and worldwide, which will provide comprehensive reference for epidemic prevention.

## 2. Materials and Methods

### 2.1. Data Collection

The complete genomic sequences of SARS-CoV-2 were obtained from 2019 Novel Coronavirus Resource (2019nCoVR) [12] and two databases, including the National Center for Biotechnology Information (NCBI) [13] and Global Initiative on Sharing All Influenza Data (GISAID) [14]. The DNA sequences of two other representative CoVs (SARS-CoV and MERS-CoV) were included for comparative analysis. The genomic information of latest SARS-CoV-2 strains is shown in Table S1.

### 2.2. Homology Analysis

The amino acid sequences of 28 proteins in SARS-CoV-2 were compared with those of SARS-CoV to analyze protein homology by using NCBI Blastp [15]. Proteins from SARS and SARS-CoV-2 were treated as homologous: identity value ≥ 65%, query coverage ≥ 95%.

### 2.3. Phylogenetic Analysis

Comparative genomic analyses of SARS-CoV-2 and SARS-CoV were performed by zpicture for the global comparison [16]. Multiple sequence alignment and the construction of phylogenetic trees of 38 CoVs were conducted using MEGA7 [17]. The evolutionary distances were calculated using the Maximum Composite Likelihood method [18].

## 3. Results

The differences and similarities of clinical characteristics between COVID-19 and SARS were summarized in Table 1.

### 3.1. COVID-19 and SARS—the Initial Events

On Nov. 27th, 2002, a respiratory illness erupted in Guangdong Province, China [19]. In Feb, 2003, the Chinese Ministry of Health announced that this acute respiratory syndrome had thus far resulted in 305 cases and five deaths [20]. The following month, there were clusters of atypical pneumonia reported in other parts of mainland China, Hong Kong [21], Canada [22], and Singapore [23]. In Jul, 2003, SARS-CoV spread across 26 countries in six continents, and caused a cumulative 8,096 cases and 774 deaths (9.6%) [24]. In particular, a higher mortality (21%) was found in hospital personnel [25,26].

**Figure 1:**
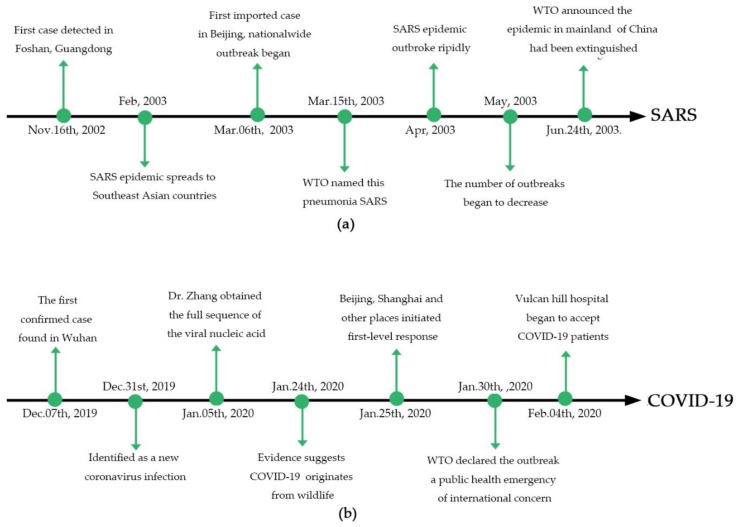
Timeline of SARS (**a**) and COVID-19 (**b**) epidemic development.

On Dec. 29th, 2019, the health departments of Hubei Province received a report that four employees of the South China Seafood Wholesale Market were diagnosed with unknown-caused pneumonia in a local hospital, which was the first report of SARS-CoV-2 [27]. On Dec. 31st, 2019, the National Health Commission of People Republic of China and Chinese Center for Disease Control and Prevention (China CDC) participated in the investigation and case-searching work [27]. On the same day, the government of Wuhan released information about the disease outbreaks to society [28]. Nowadays, the number of patients infected with SARS-CoV-2 continues to climb worldwide. By the date of this paper’s submission, a cumulative 67,081 cases and 1,526 deaths (2.1%) were reported worldwide. In Wuhan, China, the number is 37,914. The main timeline of SARS and COVID-19 epidemic development were shown in Figure 1a,b, respectively.

### 3.2. Clinical Symptoms

The initial symptoms of SARS patients were fever (100%), cough (61.8%), myalgia (48.7%), dyspnea (40.8%), and diarrhea (31.6%) [29], and the prognosis of patients was associated with host characteristics (including age, gender, etc.) [30]. During hospitalization, respiratory distress occurred in 90.8% of SARS patients [29]. The duration from disease onset to severe respiratory distress was an average of 9.8 ± 3.0 days [29]. During the disease course, some patients developed leukopenia, lymphopenia, and thrombocytopenia with an upregulation of aspartate transaminase (AST), alanine aminotransferase (ALT), lactic dehydrogenase (LDH), and C-reactive protein (CRP) [29].

In comparison, COVID-19 showed similar trends with SARS patients [28]. Fever, fatigue, and dry cough are the main manifestations of the patients, while nasal congestion, runny nose, and other symptoms of the upper respiratory tract are rare. Beijing Centers for Diseases Control and Prevention indicated that the typical case of COVID-19 has a progressive aggravation process. COVID-19 can be classified into light, normal, severe, and critical types based on the severity of the disease [31]: (1) Mild cases—the clinical symptoms were mild, and no pneumonia was found on the chest computed tomography (CT); (2) normal cases—fever, respiratory symptoms, and patients found to have imaging manifestations of pneumonia; (3) severe cases—one of the following three conditions: Respiratory distress, respiratory rate ≥ 30 times/min (in resting state, refers to oxygen saturation ≤ 93%), partial arterial oxygen pressure (PaO2)/oxygen absorption concentration (FiO2) ≤ 300 mmHg (1 mmHg = 0.133 kPa); (4) critical cases—one of the following three conditions: Respiratory failure and the need for mechanical ventilation, shock, or the associated failure of other organs requiring the intensive care unit [32]. The current clinical data shows that the majority of the deaths occurred in the older patients. However, severe cases have been documented in young adults who have unique factors, particularly those with chronic diseases, such as diabetes or hepatitis B. Those with a long-term use of hormones or immunosuppressants, and decreased immune function, are likely to get severely infected.

### 3.3. Virus Incubation

The incubation period of SARS is 1–4 days [33]. However, in a small number of patients, the incubation period may be longer than 10 days [34]. It has been demonstrated that the latency of COVID-19 varies from 3–7 days on average, for up to 14 days [35]. During this incubation period, patients are contagious, and it has been reported that each case infected on average 3.77 other people (uncertainty range 2.23-4.82) [36]. By comparison, we found that the average latency of COVID-19 is slightly longer than that of SARS.

### 3.4. Susceptible Populations

According to the demographic information of SARS patients, infection occurred in all age groups (the average age was ≦45) [37]. There was a proportional difference between male and female (female predominance) [37], with a male-to-female ratio of 1:1.25 [38]. In addition, hospital staff had a higher risk due to the proximal interactions with large numbers from the infected population. For example, hospital staff accounted for 22% of all cases in Hong Kong and 22.8% in Guangdong [37]. The mortality caused by SARS increased with age (> 64 years) [37], and the overall mortality rate during the outbreak of SARS was estimated at 9.6% [39,40].

Li et al. reported that people who have not been exposed to SARS-CoV-2 are all susceptible to COVID-19 [41]. Among the 8,866 patients who have been confirmed with COVID-19, nearly half of the patients have been aged 50 years or older (47.7%) [36]. The male-to-female ratio is about 2.7:1 [38] and the average incubation period is 5.2 days [42]. However, severe COVID-19 cases and deaths have mostly been in the middle-aged adults and the elderly with long smoking histories or other basic diseases, such as heart disease and hypertension [43,44]. At the time that this paper was been submitted, COVID-19 patients mortality rate was 2.1% [45].

### 3.5. Animal Reservoirs

In 2010, Shi et al. isolated a SARS-like coronavirus which was highly homologous to the SARS-CoV, confirming that *Rhinolophus sinicus* (Chinese rufous horseshoe bat) was a natural host of the SARS-CoV [46]. Bats are known to be hosts for 30 coronaviruses based on complete genomic sequences analysis [47]. Epidemiological investigations have shown that civet cats in the wildlife market were the direct source of SARS-CoV [48]. Among the four patients with SARS discovered during the winter of 2003-2004, two were waitresses at a restaurant in Guangzhou, China, and one was a customer who ate in the restaurant a short distance from a civet cage. All six civets in this cage were tested positive for SARS-CoV [48].

The new emerging SARS-CoV-2 shares about 80% of the gene sequence of SARS-CoV, released by the Military Medical Research Institute of Nanjing Military Region in 2003 [28]. Recently, Shi et al. reported that the sequence similarity of coronavirus between SARS-CoV-2 and the coronavirus isolated from *Rhinolophus affinis* is 96.2%, and suggested that bats may be the source of the virus [49]. So far, the intermediate hosts of SARS-CoV-2 are elusive and have been reported to be snakes, minks, or variable others [50,51]. Recently, a research group of South China Agricultural University reported that pangolins may be one of the intermediate hosts for SARS-CoV-2, by analyzing more than 1,000 metagenomic samples, because they found that 70% of pangolins are positive for the coronavirus. Moreover, the virus isolate from pangolin shared 99% sequence similarity with the current infected human strain SARS-CoV-2 [52]. Taking this recent research into consideration, we agreed that pangolin is more likely to be one of intermediate hosts of SARS-CoV-2.

### 3.6. Regional Distribution

**Figure 2:**
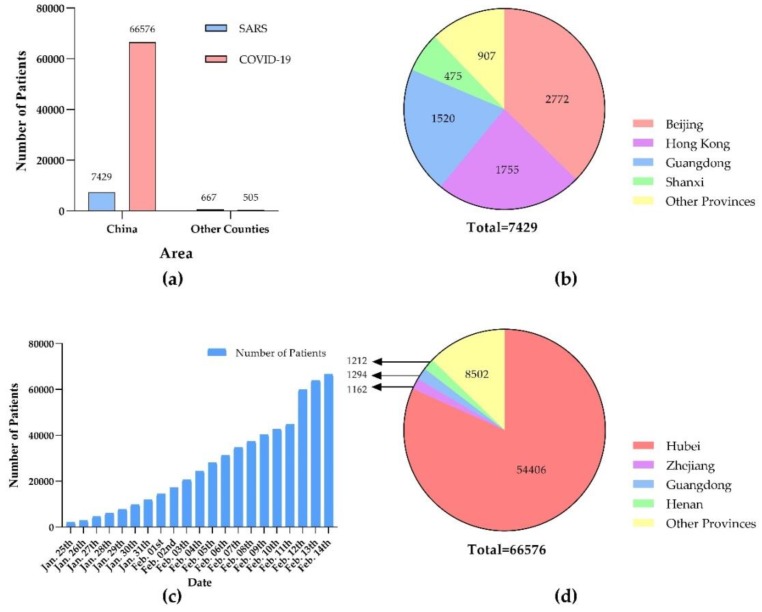
(**a**) Comparison of the number of SARS and COVID-19 patients in China (including Hong Kong, Macao and Taiwan) and other countries; (**b**) the number of SARS patients in different provinces of China; (**c**) an increased number of COVID-19 patients over time was showed in a histogram. On Feb. 11th, Hubei Province had added a “clinical diagnosis case” classification, and identified suspected cases with pneumonia imaging features as clinical diagnosis cases so that patients can receive standardized treatment as soon as possible. (**d**) The number of COVID-19 patients in different provinces of China. The time period shown in the picture was in the Spring Festival transportation.

According to the WHO data on Jul. 31th, 2003 [24], a total of 8,096 clinically diagnosed cases of SARS were reported worldwide, with 774 deaths and 26 countries and regions affected (Figure 2a). Most cases were in Asia, Europe, and America. The main countries in Asia were China (including mainland, Macao, Hong Kong, and Taiwan), Singapore, and so on. The total number of cases in mainland China was 5,327, with 349 deaths [53]. The cases were mainly concentrated in Beijing, Guangdong, and Shanxi (Figure 2b) [54]. In total, 2,102 patients were from Hong Kong, Macao, and Taiwan, with 336 deaths [24]. 

**Figure 3:**
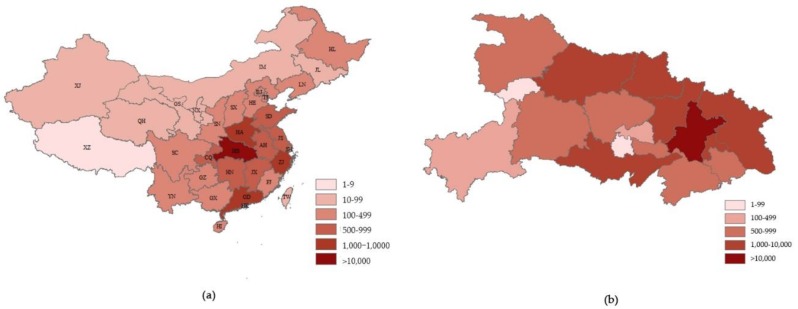
The distribution of COVID-2019 patients in China (**a**) and Hubei Province (**b**). XJ, Xinjiang; XZ, Xizang; GS, Gansu; QH, Qinghai; SC, Sichuan; YN, Yunnan; IM, Inner Mongolia; NX, Ningxia; SN, Shaanxi; CQ, Chongqing; GZ, Guizhou; GX, Guangxi; HI, Hainan; SX, Shanxi, HA, Henan; HB, Hubei; HN, Hunan; GD, Guangdong; HK, Hong Kong; HE, Hebei; BJ, Beijing; TJ, Tianjin; SD, Shandong; AH, Anhui; JX, Jiangxi; JS, Jiangsu; SH, Shanghai; ZJ, Zhejiang; FJ, Fujian; TW, Taiwan; HL, Heilongjiang; JL, Jilin; LN, Liaoning.

According to the latest data on Feb. 14th, 2020 [55,56], there have been a total of 67,081 clinically diagnosed cases of COVID-19 in worldwide, with 1,526 deaths. A total of 25 countries and regions have infected people. Due to the Spring Festival transportation peak, the disease has been spread more rapidly across China (Figure 2c). As the origin area of COVID-19, Hubei province has been the most severely infected area, with 54,406 cumulative diagnosis cases. Wuhan city has 37,914 cases. Guangdong, Henan, and Zhejiang province have 1,294 cases, 1,212 cases, and 1,162 cases, respectively (Figure 2d). At present, the COVID-19 outbreak has been spread to all parts of China and around the world, including the United States, Thailand, and Japan. It has been noticed that most of these patients have ever been to Wuhan or contacted with people who had been in Wuhan. The distribution of COVID-2019 patients in China (including Hong Kong, Macao and Taiwan) and Hubei Province is shown in Figure 3. 

### 3.7. Prevention, Diagnosis, and Treatment

#### 3.7.1. Prevention

As the number of COVID-19 patients in China has been growing rapidly, preventing the spread of SARS-CoV-2 is the most important and urgent task [57]. It was shown that human-to-human transmission of SARS-CoV-2 has spread via droplets or close contacts [58,59], but aerosol and fecal-oral transmission still need further study [60,61]. To reduce virus transmission, early detection and isolation are essential. In addition, close monitoring in crowded places is also important [62]. The possible pathogens of SARS and COVID-19 are both derived from wild animals [63]. Therefore, hunting, selling, and eating wild animals not only seriously damage the ecosystem, but also lead to the spread of epidemic diseases [64]. Thus, banning all wildlife trade is an effective measure to prevent viral prevalence. Wearing level-D protective clothing can protect medical staff from infection of respiratory viruses [65]. A vaccine against SARS-CoV has not been described in any published articles [66]. However, on Jan. 26th, 2020, the China CDC started to develop a new vaccine for SARS-CoV-2. The virus has been successfully isolated and seed strains have been screened [67].

#### 3.7.2. Diagnosis

The early symptoms of SARS and COVID-19 are very similar to winter influenza, and the most important way to distinguish flu and pneumonia is to take throat swabs for viral testing [68]. Current diagnostic tests for coronavirus include RT-PCR, real-time reverse transcription PCR (rRT-PCR), reverse transcription loop-mediated isothermal amplification, as well as real-time RT-LAMP [69,70,71,72]. National Medical Products Administration has approved seven new nucleic acid test reagents for coronavirus, which were developed based on fluorescence PCR by Feb. 1st, 2020 [73]. Suspected infections can be detected accurately and quickly for timely isolation and treatment to avoid infecting others by using these test reagents.

#### 3.7.3. Treatment

Both SARS-CoV and SARS-CoV-2 are CoVs; hence, the treatment strategies of SARS could be relevant for COVID-19 [74]. In 2003, SARS was mainly treated by isolation of the patients, hormones treatment, antiviral and symptomatic treatments, and many drugs such as glucocorticoid [29] and interferon [75]. Now, isolation, antiviral, and symptomatic treatments are still mainly adopted for COVID-19 treatment. As effective drugs for SARS, hormones and interferons can also be used to treat COVID-19 [74]. Lopinavir is one kind of protease inhibitor used to treat HIV infection, with ritonavir as a booster. Lopinavir and/or ritonavir has anti coronavirus activity in vitro. Hong Kong scholars found that, compared with ribavirin alone, patients treated with lopinavir/ritonavir and ribavirin had lower risk of acute respiratory distress syndrome (ARDS) or death caused by SARS-CoV [76,77]. Lopinavir/ritonavir has also been clinically tested in treatment of COVID-19, and showed wonderfully effective treatment for some patients, but the general clinical effect has not been determined [78].

More effective treatments are still under continuing exploration: On Jan. 25th, 2020, a joint research team from the Shanghai Institute of Materia Medica, Chinese Academy of Sciences, and Shanghai Tech University screened and identified 30 potential drugs that are reported to be effective against SARS-CoV-2 [79]. A high-resolution crystal structure of SARS-CoV-2 coronavirus 3CL hydrolase (Mpro) was announced after the outbreak of COVID-19 in the world [80], and human coronaviruses (HCoVs) have been treated as severe pathogens in respiratory tract infections. Nelfinavir was predicted to be a potential inhibitor of SARS-CoV-2 main protease [81]. The first patient in the US had been trial-treated with intravenous remdesivir (a novel nucleotide analogue prodrug in development) due to a severe infection [82,83]. No adverse reactions were observed during the administration, and the patient’s condition was effectively improved [84]. Clinical trials of remdesivir for treatment of COVID-19 just started on Feb. 5th and 12th, 2020 in Wuhan and Beijing, respectively, and the experimental results remain unclear [85,86].

### 3.8. Genomic Comparison

CoVs are RNA viruses and contain the largest genomes of all RNA viruses [87]. CoVs belong to the subfamily Coronavirinae in the family of Coronaviridae of the order Nidovirales, and this subfamily includes four genera: α-coronavirus, β-coronavirus, γ-coronavirus, and δ-coronavirus [88]. Both SARS-CoV-2 and SARS-CoV are in the coronavirus family, β-coronavirus genera [89]. The genome of SARS-CoV-2 is more than 85% similar to the genome of the SARS-like virus ZC45 (bat-SL-CoVZC45, MG772933.1), and together these types of viruses form a unique Orthocoronavirinae subfamily with another SARS-like virus ZXC21 in the sarbecovirus subgenus [35]. All the three viruses show typical β-coronavirus gene structure. Human SARS-CoV and a genetically similar bat coronavirus (bat-SL-CoVZXC21, MG772934) from southwest of China have formed another clade within the sarbecovirus [35].

**Figure 4:**
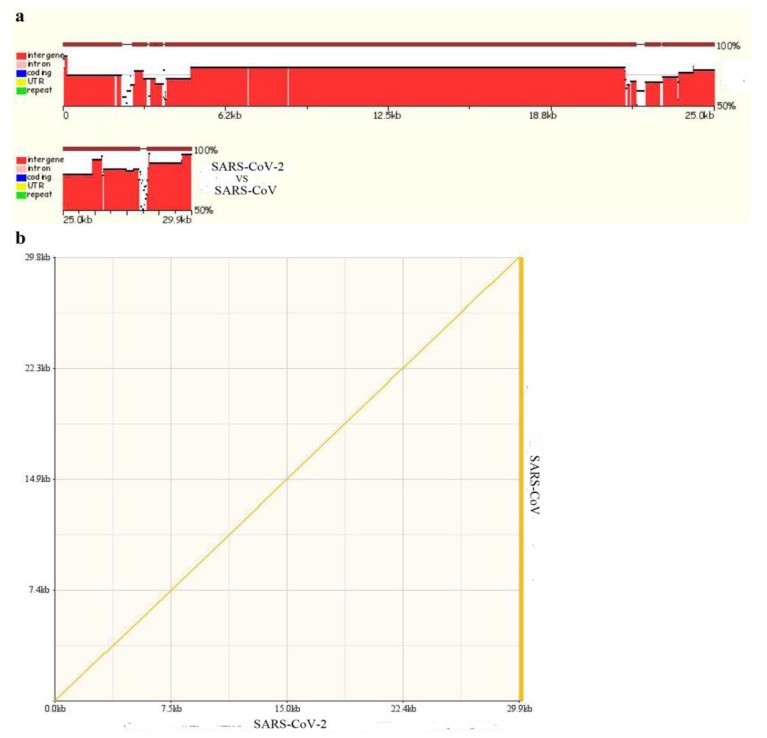
(**a**) Genomic sequence alignment between SARS-CoV-2 and SARS-CoV; (**b**) Dot plot matrix calculated for the complete genomes of SARS-CoV-2 and SARS-CoV.

We also performed comparative genomic analyses of SARS-CoV-2 and SARS-CoV by zpicture. The results showed that the genomic sequences of SARS-CoV-2 and SARS-CoV have extremely high homology at the nucleotide level (Figure 4a,b). There are six regions of difference (RD) in the genome sequence between SARS-CoV and SARS-CoV-2, and the RDs are named according to the order of discovery. RD1, RD2, and RD3 (448nt, 55nt, and 278nt, respectively) are partial coding sequences of the *orf lab* gene; RD4 and RD5 (315nt and 80nt, respectively) are partial coding sequences of the *S* gene; RD6 is 214nt in size and is part of the coding sequence of the *orf7b* and *orf8* genes. These RDs may provide new molecular markers for the identification of SARS-CoV-2 and SARS-CoV, and also help to develop new drugs against SARS-CoV-2.

To analyze the homogeneity of SARS-CoV, MERS-CoV, and SARS-CoV-2, an evolutionary tree was constructed based on the genomes of 35 SARS-CoV-2 strains from different data submission units, one SARS-CoV strain, and two MERS-CoV strains (Figure S1). Phylogenetic analysis showed that the distance of SARS-CoV (AY274119) is closer to SARS-CoV-2 strains than MERS-CoV (KC164505, JX869059).

### 3.9. Proteomic Comparison

To further explore whether all encoded proteins of SARS-CoV-2 are homologous to that of SARS-CoV, we performed a protein sequence alignment analysis using Blastp. The results showed that most of SARS-CoV-2 proteins are highly homologous (95%–100%) to the proteins of SARS-CoV virus, indicating the evolutionary similarity between SARS-CoV and SARS-CoV-2 (Table 2). However, two proteins (orf8 and orf10) in SARS-CoV-2 have no homologous proteins in SARS-CoV. The amino acid sequence of orf8 in SARS-CoV-2 is different from sequences of conserved orf8 or orf8b derived from human SARS-CoV [11]. Orf8 protein of SARS-CoV-2 does not contain known functional domain or motif. An aggregation motif VLVVL (amino acid 75–79) has been found in SARS-CoV orf8b which was shown to trigger intracellular stress pathways and activate NOD-like receptor family pyrin domain-containing-3 (NLRP3) inflammasomes [90]. Therefore, it will be clinically meaningful to analyze the biological function of these two specific proteins (orf8 and orf10) in SARS-CoV-2.

As the most abundant protein in CoVs, nucleocapsid (N) protein is highly conserved across CoVs [91]. The N protein in SARS-CoV-2 shares ~90% amino acid identity with that in SARS-CoV [28], which indicates that antibodies against the N protein of SARS-CoV would likely recognize and bind the N protein of SARS-CoV-2 as well. N antibodies do not provide immunity to SARS-CoV-2 infection, but the antibodies have a cross reactivity with SARS-CoV N protein viruses, which would allow a serum-based assay to identify the asymptomatic SARS-CoV-2 infected-cases [28]. Although previous studies have found serum reactivity to group SARS-CoV N proteins in Chinese populations [92], exposure to SARS-CoV-2 should increase the dilution factor if the infection had occurred. This information has important implications for preventing the spread of asymptomatic infections.

In addition, spike stalk S2 in SARS-CoV-2 is highly conserved and shares 99% identity with those of the two bat SARS-like CoVs (bat-SL-CoVZXC21 and bat-SL-CoVZC45) and human SARS-CoV [11]. Thus, the broad spectrum antiviral peptides against S2 has the potential to be effective treatment [93].

### 3.10. Pathogenic Mechanisms

Many studies have been performed to study the pathogenesis of SARS-CoV [94,95,96]. The spike (S) protein and N protein confer stability to the viral particle [97]. The N protein is a structural protein involved in virion assembly, and plays a pivotal role in virus transcription and assembly efficiency [97]. S protein can bind to the cellular receptors of sensitive cells and mediate infection of their target cells, after which it begins to replicate in the cytoplasm [98]. SARS-CoV mainly targets the lungs, immune organs, and small systemic blood vessels and causes systemic vasculitis and decrease of immune function [99]. More seriously, the infection leads to extensive pulmonary consolidation, diffuse alveolar damage, and the formation of a transparent membrane, finally deteriorating to respiratory distress [100].

CoV can enter host cells through the interaction between CoV S protein and its host receptor angiotensin-converting enzyme 2 (ACE2), which is isolated from SARS-CoV–permissive Vero-E6 cells. The receptor-binding motif (RBM) of S protein can directly contact ACE2 [47,101,102]. AEC2 have been identified to be key binding residues and functional receptor for SARS-CoV, and it can also protect alveolar cells [103]. The binding of spike protein to ACE2 and the subsequent downregulation of this receptor contribute to severe alveolar injury during SARS [49]. The downregulation of ACE2 results in the excessive production of angiotensin II by the related enzyme ACE, and the stimulation of type 1A angiotensin II receptor (AGTR1A) can lead to the increase of pulmonary vascular permeability, which potentially explains the increased lung pathology when the expression of ACE2 is decreased [104]. ACE2-transfected T cells form multinucleated syncytia with cells expressing S protein. The virus was shown to replicate effectively in ACE2-transfected, but not in mock-transfected T cells. Antibodies targeting ACE2 can block viral replication in Vero-E6 cells [105].

Recently, Ji et al. demonstrated that the receptor binding domain of SARS-CoV-2 was capable of binding ACE2 in the context of the SARS-CoV spike protein [51]. Among SRAS-CoV spike protein’s fourteen residues predicted to interact directly with human ACE2 as the receptor for SARS-CoV, eight amino acids are well conserved in homology SARS-CoV-2 spike protein [26,102]. At the same time, Wan et al. showed that SARS-CoV-2 uses ACE2 receptors to infect humans, bats, civets, monkeys, and swine, but not mice [106]. Compared to previously reported SARS-CoV strains, SARS-CoV-2 uses ACE2 receptors more efficiently than human SARS-CoV (year 2003), but less efficiently than human SARS-CoV (year 2002) [106]. The mutation of proteins determines two important characteristics of the SARS-CoV-2: A higher ability to infect and enhanced pathogenicity than the bat-like SARS-CoV, but a lower pathogenicity than SARS-CoV [43].

## 4. Discussion

As a large number of people have left Wuhan, the control of the epidemic situation is extremely urgent, and the treatments of COVID-19 are imminent. On Feb. 14th, 2020, there were more than 54,000 confirmed patients in Hubei province, China [55]. Due to the lack of effective antiviral drugs, the prognosis of patients solely depends on their age and physical condition [74]. Although it was reported that the clinically recovered patients exceed the number of dead, the majority of the patients are still not cured in hospital. In addition, the potential adaptive mutation of SARS-CoV-2 makes it difficult for vaccine development. Therefore, it is urgent for us to develop more sensitive inspection methods and effective drugs.

Seven type of CoVs have been identified to cause human disease [107]. The two highly pathogenic viruses, SARS-CoV and MERS-CoV, cause severe respiratory syndrome in humans. The other four human CoVs (HCoV-NL63, HCoV-229E, HCoV-OC43 and HKU1) induce only mild upper respiratory diseases, although some of them can cause severe infections in infants, young children, and elderly individuals [4,108]. The latest one is SARS-CoV-2. It has been reported that SARS-CoV-2 shared almost 80% of the genome with SARS-CoV [11]. Our results also showed that almost all encoded proteins of SARS-CoV-2 are homologous to SARS-CoV proteins (Table 2). Hence, clinical drugs and therapies for treating SARS may be used as a reference for COVID-19 treatment [74].

In addition to the well-known SARS-CoV, MERS-CoV, as one Merbecovirus subgenus of β-CoVs, is also extremely invasive. MERS-CoV is the pathogen of the Middle East Respiratory Syndrome, which can infect both humans and animals, and can be transmitted through camels [109]. It mainly occurs in Saudi Arabia and has a high mortality rate [66]. Studies had demonstrated that the clinical course of SARS and MERS was highly similar, and SARS and MERS may have similar pathogenesis [66]. The genome sequence of SARS-CoV-2 also shows some similarities to that of MERS-CoV. It will be very interesting to study the relationship among SARS-CoV, MERS-CoV, and SARS-CoV-2 that may be exploited for future developing broad-spectrum antiviral therapies.

Although more and more studies for SARS-CoV-2 have sprung up since the outbreak of this epidemic COVID-19, based on our comparison, we propose some key questions to be clarified in future studies (Table 3). In-depth understanding the underlying pathogenic mechanisms of SARS-CoV-2 will reveal more targets for better therapy of COVID-19.

## Figures and Tables

**Table 1:** Comparison of SARS and COVID-19.

Items	SARS	COVID-19
First occurrence	Nov. 16th, 2002 in Foshan, Guangdong	Dec. 07th, 2019 in Wuhan, Hubei
Pathogen	SARS-CoV	SARS-CoV-2
Intermediate host	*Paguma larvata*	Pangolin, Mink (Possible)
Definitive host	*Rhinolophus sinicus*	*Rhinolophus affinis* (Possible)
Virus type	RNA virus	RNA virus
Species pathogen	β-coronavirus	β-coronavirus
Total DNA sequence length of pathogen	29,751	29,903
Latency	1–4 days on average	3–7 days on average
Susceptible people	Young adults	People who have not been exposed to SARS-CoV-2
Male–female patient ratio	1:1.25	2.70:1
Mortality	9.60%	2.10%
Clinical symptoms	Fever, cough, myalgia, dyspnea, and diarrhea	Fever, fatigue, and dry cough
Propagation mode	Droplets or close contacts	Droplets or close contacts
Major regional distribution	Beijing, Guangdong, Shanxi in China	Hubei, especially Wuhan in China
Diagnostic methods	RT-PCR, rRT-PCR, RT-LAMP, rRT-LAMP, Coronavirus detection kit	RT-PCR, rRT-PCR, RT-LAMP, rRT-LAMP, Coronavirus detection kit
Treatment	Glucocorticoid and interferon	Lopinavir/ritonavir (in testing)

**Table 2:** Comparison of protein sequences SARS-CoV-2 and SARS-CoV by Blastp.

	SARS-CoV-2		SARS-CoV			
	Protein Name	Accession Number	Putative Function/Domain	Accession Number	Query Cover *	Percent Identity
1	nsp2	YP_009725298.1	nonstructural polyprotein pp1a	ABF65834.1	100%	68.34%
2	nsp3	YP_009725299.1	polyprotein orf1a	AFR58698.1	100%	75.82%
3	nsp4	YP_009725300.1	polyprotein 1a	ARO76381.1	100%	80.80%
4	nsp6	YP_009725302.1	nsp6-pp1a/pp1ab (TM3)	NP_828864.1	98%	88.15%
5	nsp7	YP_009725303.1	Chain A, Replicase Polyprotein 1ab, Light Chain	2AHM_A	100%	98.80%
6	nsp8	YP_009725304.1	Chain E, Replicase Polyprotein 1ab, Heavy Chain	2AHM_E	100%	97.47%
7	nsp9	YP_009725305.1	nsp9-pp1a/pp1ab	NP_828867.1	100%	97.35%
8	nsp10	YP_009725306.1	Chain A, Non-structural Protein 10	5C8S_A	100%	97.12%
9	nsp11	YP_009725312.1	nsp11-pp1a	NP_904321.1	100%	84.62%
10	orf1a polyprotein	YP_009725295.1	orf1a polyprotein (pp1a)	NP_828850.1	100%	80.58%
11	orf1ab polyprotein	YP_009724389.1	orf1ab polyprotein (pp1ab)	NP_828849.2	100%	86.26%
12	orf3a protein	YP_009724391.1	hypothetical protein sars3a	NP_828852.2	100%	72.04%
13	orf6 protein	YP_009724394.1	hypothetical protein sars6	NP_828856.1	100%	68.85%
14	orf7a protein	YP_009724395.1	protein 8	ARO76387.1	100%	87.70%
15	orf7b protein	YP_009725296.1	hypothetical protein sars7b	NP_849175.1	95%	85.37%
16	orf8 protein	YP_009724396.1	-	-	-	-
17	orf10 protein	YP_009725255.1	-	-	-	-
18	2’-O-ribose methyltransferase	YP_009725311.1	nsp16-pp1ab (2’-o-MT)	NP_828873.2	99%	93.60%
19	3C-like proteinase	YP_009725301.1	polyprotein 1a	ARO76381.1	100%	96.08%
20	3’-to-5’ exonuclease	YP_009725309.1	nsp14-pp1ab (nuclease ExoN homolog)	NP_828871.1	100%	95.07%
21	endoRNAse	YP_009725310.1	nsp15-pp1ab (endoRNAse)	NP_828872.1	100%	88.73%
22	envelope protein	YP_009724392.1	E protein	APO40581.1	100%	94.74%
23	helicase	YP_009725308.1	nsp13-pp1ab (ZD, NTPase/HEL)	NP_828870.1	100%	99.83%
24	leader protein	YP_009725297.1	nsp1-pp1a/pp1ab	NP_828860.2	100%	84.44%
25	membrane glycoprotein	YP_009724393.1	matrix protein	NP_828855.1	100%	90.54%
26	nucleocapsid phosphoprotein	YP_009724397.2	nucleocapsid protein	ARO76389.1	100%	90.52%
27	RNA-dependent RNA polymerase	YP_009725307.1	nsp12-pp1ab (RdRp)	NP_828869.1	100%	96.35%
28	surface glycoprotein	YP_009724390.1	spike glycoprotein	ABD72985.1	100%	76.42%

“-” represents no homologous protein. Query cover represents the percentage of the protein sequences that are participating in the comparison. Percent identity indicates the homology.

**Table 3:** Proposed questions to study SARS-CoV-2 for future studies.

What is the effect of the surface epitope and receptor binding domain of S protein of SARS-CoV-2 on the virus’ infectivity?
Is there any effect of SARS-CoV vaccine designed according to S protein on SARS-CoV-2?
Does SARS-CoV-2 orf8 and orf10 proteins, which have no homology proteins in SARS-CoV, play roles in the infectivity and pathogenicity of SARS-CoV-2?
Can the susceptibility of asymptomatic carriers be judged by detecting the serum reactivity level of N protein?
Apart from droplet transmission and contact transmission, are there other methods to transmit SARS-CoV-2?
What is the percentage of COVID-19 patients have been infected with SARS and produced antibodies?
Is there an effective specific anti-SARS-CoV-2 solution?
Does traditional Chinese medicine have any effect on the treatment of COVID-19 caused by SARS-CoV-2?
Do ethnic differences affect the transmissibility and pathogenicity of SARS-CoV-2?
Do any environmental factors, such as regional conditions or climate, affect SARS-CoV-2 transmission?

## Supplementary Materials

The following are available online at https://www.mdpi.com/1999-4915/12/2/244/s1, Figure S1: Phylogeny of SARS-CoV-2, SARS-CoV and MERS-CoV. Unrooted phylogenetic relationships are represented by constructing Neighbor-Joining tree. Table S1: The genomic information of latest SARS-CoV-2 strains
