# Supplementary material for: Systematic Comparison of Two Animal-to-Human Transmitted Human Coronaviruses: SARS-CoV-2 and SARS-CoV
Source: Viruses. 2020 Feb 22;12(2):244. doi: 10.3390/v12020244 (PMC7077191; doi:10.3390/v12020244)
Supplement: Supplementary file 1 [file viruses-12-00244-s001.zip › Table S1.docx]

**Table S1.** The genomic information of latest SARS-CoV-2 strains.

| Accession number | Virus name | Region | Year |
| --- | --- | --- | --- |
| EPI_ISL_410218  EPI_ISL_410301 | BetaCov/Taiwan/NTU02/2020  BetaCoV/Nepal/61/2020 | China / Taiwan / Taipei  Nepal / Kathmandu | 2020  2020 |
| MT039890 | SNU01 | South Korea | 2020 |
| MT049951 | SARS-CoV-2/Yunnan-01/human/2020/CHN | China | 2020 |
| EPI_ISL_410044 | BetaCoV/USA/CA6/2020 | USA / California | 2020 |
| EPI_ISL_410045 | BetaCoV/USA/IL2/2020 | USA / Illinois | 2020 |
| EPI_ISL_408976 | BetaCoV/Sydney/2/2020 | Australia / New South Wales / Sydney | 2020 |
| EPI_ISL_408977 | BetaCoV/Sydney/3/2020 | Australia / New South Wales / Sydney | 2020 |
| EPI_ISL_408482 | BetaCoV/Shandong/IVDC-SD-001/2020 | China / Shandong / Qingdao | 2020 |
| LC522973 | 2019-nCoV/Japan/TY/WK-012/2020 RNA | Japan / Tokyo | 2020 |
| LC522974 | 2019-nCoV/Japan/TY/WK-501/2020 | Japan / Tokyo | 2020 |
| LC522975 | 2019-nCoV/Japan/TY/WK-521/2020 | Japan / Tokyo | 2020 |
| EPI_ISL_408668 | BetaCoV/Vietnam/VR03-38142/2020 | Vietnam / Thanh Hoa | 2020 |
| LC522972 | 2019-nCoV/Japan/KY/V-029/2020 | Japan / Kyoto | 2020 |
| MT039887 | 2019-nCoV/USA-WI1/2020 | USA / Wisconsin | 2020 |
| EPI_ISL_408478 | Chongqing/YC01/2020 | China / Chongqing / Yongchuan | 2020 |
| EPI_ISL_408484 | Sichuan/IVDC-SC-001/2020 | China / Sichuan / Chengdu | 2020 |
| EPI_ISL_408480 | Yunnan/IVDC-YN-003/2020 | China / Yunnan / Kunming | 2020 |
| EPI_ISL_408489 | Taiwan/NTU01/2020 | China / Taiwan / Taipei | 2020 |
| EPI_ISL_408515 | Wuhan/IVDC-HB-envF13-21/2020 | China / Hubei / Wuhan | 2020 |
| EPI_ISL_408431 | France/IDF0626/2020 | France / Ile De France / Paris | 2020 |
| EPI_ISL_408486 | Jiangxi/IVDC-JX-002/2020 | China / Jiangxi / Pingxiang | 2020 |
| EPI_ISL_408479 | Chongqing/ZX01/2020 | China / Chongqing / Zhongxian | 2020 |
| EPI_ISL_408488 | Jiangsu/IVDC-JS-001/2020 | China / Jiangsu / Huaian | 2020 |
| EPI_ISL_408430 | France/IDF0515/2020 | France / Ile De France / Paris | 2020 |
| EPI_ISL_408481 | Chongqing/IVDC-CQ-001/2020 | China / Chongqing | 2020 |
| EPI_ISL_408514 | Wuhan/IVDC-HB-envF13-20/2020 | China / Hubei / Wuhan | 2020 |
| MT027062 | 2019-nCoV/USA-CA3/2020 | USA / California | 2020 |
| MT027063 | 2019-nCoV/USA-CA4/2020 | USA / California | 2020 |
| MT027064 | 2019-nCoV/USA-CA5/2020 | USA / California | 2020 |
| EPI_ISL_407894 | BetaCoV/Australia/QLD01/2020 | Australia / Queensland / Gold Coast | 2020 |
| EPI_ISL_407896 | BetaCoV/Australia/QLD02/2020 | Australia / Queensland / Gold Coast | 2020 |
| EPI_ISL_407893 | BetaCoV/Australia/NSW01/2020 | Australia / New South Wales / Sydney | 2020 |
| EPI_ISL_407976 | BetaCoV/Belgium/GHB-03021/2020 | Belgium / Leuven | 2020 |
| EPI_ISL_407988 | BetaCoV/Singapore/3/2020 | Singapore | 2020 |
| EPI_ISL_407987 | BetaCoV/Singapore/2/2020 | Singapore | 2020 |
| EPI_ISL_407313 | BetaCoV/Hangzhou/HZCDC0001/2020 | China / Zhejiang / Hangzhou | 2020 |
| MT020881 | BetaCoV/USA/WA1-F6/2020 | USA / Washington | 2020 |
| EPI_ISL_407193 | BetaCoV/Korea/KCDC03/2020 | Korea / Gyeonggi-do | 2020 |
| LC521925 | BetaCoV/Japan/AI/I-004/2020 | Japan / Aichi | 2020 |
| MT020880 | BetaCoV/USA/WA1-A12/2020 | USA / Washington | 2020 |
| EPI_ISL_406973 | BetaCoV/Singapore/1/2020 | Singapore | 2020 |
| EPI_ISL_407073 | BetaCoV/England/02/2020 | England | 2020 |
| EPI_ISL_407071 | BetaCoV/England/01/2020 | England | 2020 |
| GWHABKS00000000 | 20cov-1L | China / Zhejiang / Hangzhou | 2020 |
| NMDC60013002-05 | BetaCoV/Wuhan/WH19002/2019 | China / Hubei Province / Wuhan City | 2019 |
| NMDC60013002-06 | BetaCoV/Wuhan/WH19008/2019 | China / Hubei Province / Wuhan City | 2019 |
| NMDC60013002-07 | BetaCoV/Wuhan/YS8011/2020 | China / Hubei Province / Wuhan City | 2020 |
| NMDC60013002-08 | BetaCoV/Wuhan/WH19001/2019 | China / Hubei Province / Wuhan City | 2019 |
| NMDC60013002-09 | BetaCoV/Wuhan/WH19004/2020 | China / Hubei Province / Wuhan City | 2020 |
| NMDC60013002-10 | BetaCoV/Wuhan/WH19005/2019 | China / Hubei Province / Wuhan City | 2019 |
| EPI_ISL_406862 | BetaCoV/Germany/BavPat1/2020 | Germany / Bavaria / Munich | 2020 |
| NMDC60013002-01 | BetaCoV/Wuhan/WH-01/2019 | China / Hubei Province / Wuhan City | 2019 |
| NMDC60013002-03 | BetaCoV/Wuhan/WH-03/2019 | China / Hubei Province / Wuhan City | 2020 |
| NMDC60013002-04 | BetaCoV/Wuhan/WH-04/2019 | China / Hubei Province / Wuhan City | 2020 |
| MT007544 | BetaCoV/Australia/VIC01/2020 | Australia / Victoria / Clayton | 2020 |
| GWHABKK00000000 | WIV02 | China/ Hubei / Wuhan | 2019 |
| GWHABKL00000000 | WIV04 | China / Hubei / Wuhan | 2019 |
| GWHABKM00000000 | WIV05 | China / Hubei / Wuhan | 2019 |
| GWHABKN00000000 | WIV06 | China / Hubei / Wuhan | 2019 |
| GWHABKO00000000 | WIV07 | China / Hubei / Wuhan | 2019 |
| GWHABKP00000000 | TG13 | China / Yunnan / Pu'er | 2013 |
| EPI_ISL_406531 | BetaCoV/Guangdong/20SF174/2020 | China / Guangdong Province | 2020 |
| EPI_ISL_406533 | BetaCoV/Guangzhou/20SF206/2020 | China / Guangdong Province / Guangzhou City | 2020 |
| EPI_ISL_406534 | BetaCoV/Foshan/20SF207/2020 | China / Guangdong Province | 2020 |
| EPI_ISL_406535 | BetaCoV/Foshan/20SF210/2020 | China / Guangdong Province | 2020 |
| EPI_ISL_406536 | BetaCoV/Foshan/20SF211/2020 | China / Guangdong Province | 2020 |
| EPI_ISL_406538 | BetaCoV/Guangdong/20SF201/2020 | China / Guangdong Province | 2020 |
| EPI_ISL_406592 | BetaCoV/Shenzhen/SZTH-001/2020 | China/ Guangdong Province / Shenzhen City | 2020 |
| EPI_ISL_406594 | BetaCoV/Shenzhen/SZTH-003/2020 | China/ Guangdong Province / Shenzhen City | 2020 |
| EPI_ISL_406595 | BetaCoV/Shenzhen/SZTH-004/2020 | China/ Guangdong Province / Shenzhen City | 2020 |
| EPI_ISL_406593 | BetaCoV/Shenzhen/SZTH-002/2020 | China/ Guangdong Province / Shenzhen City | 2020 |
| EPI_ISL_406597 | BetaCoV/France/IDF0373/2020 | France / Ile-de-France / Paris | 2020 |
| EPI_ISL_406596 | BetaCoV/France/IDF0372/2020 | France / Ile-de-France / Paris | 2020 |
| EPI_ISL_406031 | BetaCoV/Taiwan/2/2020 | China / Taiwan / Kaohsiung | 2020 |
| MN994467 | 2019-nCoV/USA-CA1/2020 | USA / California / Los Angeles | 2020 |
| MN994468 | 2019-nCoV/USA-CA2/2020 | USA / California / Orange County | 2020 |
| MN997409 | 2019-nCoV/USA-AZ1/2020 | USA / Arizona / Phoenix | 2020 |
| MN988668 | 2019-nCoV WHU01 | China / Hubei / Wuhan | 2020 |
| MN988669 | 2019-nCoV WHU02 | China / Hubei / Wuhan | 2020 |
| MN985325 | 2019-nCoV/USA-WA1/2020 | USA / Washington / Snohomish County | 2020 |
| MN988713 | 2019-nCoV/USA-IL1/2020 | USA / Illinois /Chicago | 2020 |
| GWHABKF00000000 | BetaCoV/Wuhan/IPBCAMS-WH-01/2019 | China / Hubei Province / Wuhan City | 2019 |
| GWHABKG00000000 | BetaCoV/Wuhan/IPBCAMS-WH-02/2019 | China / Hubei Province / Wuhan City | 2019 |
| GWHABKH00000000 | BetaCoV/Wuhan/IPBCAMS-WH-03/2019 | China / Hubei Province / Wuhan City | 2019 |
| GWHABKI00000000 | BetaCoV/Wuhan/IPBCAMS-WH-04/2019 | China / Hubei Province / Wuhan City | 2019 |
| GWHABKJ00000000 | BetaCoV/Wuhan/IPBCAMS-WH-05/2020 | China / Hubei Province / Wuhan City | 2020 |
| MN938384 | 2019-nCoV_HKU-SZ-002a_2020 | China / Guangdong / Shenzhen | 2020 |
| MN975262 | 2019-nCoV_HKU-SZ-005b_2020 | China / Guangdong / Shenzhen | 2020 |
| EPI_ISL_403937 | BetaCoV/Guangdong/20SF040/2020 | China / Guangdong Province / Zhuhai City | 2020 |
| EPI_ISL_403936 | BetaCoV/Guangdong/20SF028/2020 | China / Guangdong Province / Zhuhai City | 2020 |
| EPI_ISL_403935 | BetaCoV/Guangdong/20SF025/2020 | China / Guangdong Province / Shenzhen City | 2020 |
| EPI_ISL_403934 | BetaCoV/Guangdong/20SF014/2020 | China / Guangdong Province / Shenzhen City | 2020 |
| EPI_ISL_403933 | BetaCoV/Guangdong/20SF013/2020 | China / Guangdong Province / Shenzhen City | 2020 |
| EPI_ISL_403932 | BetaCoV/Guangdong/20SF012/2020 | China / Guangdong Province / Shenzhen City | 2020 |
| EPI_ISL_404227 | BetaCoV/Zhejiang/WZ-01/2020 | China / Zhejiang Province | 2020 |
| EPI_ISL_404228 | BetaCoV/Zhejiang/WZ-02/2020 | China / Zhejiang Province | 2020 |
| EPI_ISL_402132 | BetaCoV/Wuhan/HBCDC-HB-01/2019 | China/Hubei Province | 2019 |
| EPI_ISL_403963 | BetaCoV/Nonthaburi/74/2020 | Thailand/ Nonthaburi Province | 2020 |
| EPI_ISL_403962 | BetaCoV/Nonthaburi/61/2020 | Thailand/ Nonthaburi Province | 2020 |
| EPI_ISL_402120 | BetaCoV/Wuhan/IVDC-HB-04/2020 | China / Hubei Province / Wuhan City | 2020 |
| EPI_ISL_402119 | BetaCoV/Wuhan/IVDC-HB-01/2019 | China / Hubei Province / Wuhan City | 2019 |
| EPI_ISL_402121 | BetaCoV/Wuhan/IVDC-HB-05/2019 | China / Hubei Province / Wuhan City | 2019 |
| NC_045512 | Wuhan-Hu-1 | China / Hubei Province / Wuhan City | 2019 |
| AY274119 | Severe acute respiratory syndrome-related  coronavirus isolate Tor2 | Canada | 2003 |
| JX869059 | Human betacoronavirus 2c EMC/2012 | Saudi Arabia | 2012 |
| KC164505 | Betacoronavirus England 1 | England | 2012 |
