# Supplementary figures and images for: Systematic Comparison of Two Animal-to-Human Transmitted Human Coronaviruses: SARS-CoV-2 and SARS-CoV
Source: Viruses. 2020 Feb 22;12(2):244. doi: 10.3390/v12020244 (PMC7077191; doi:10.3390/v12020244)

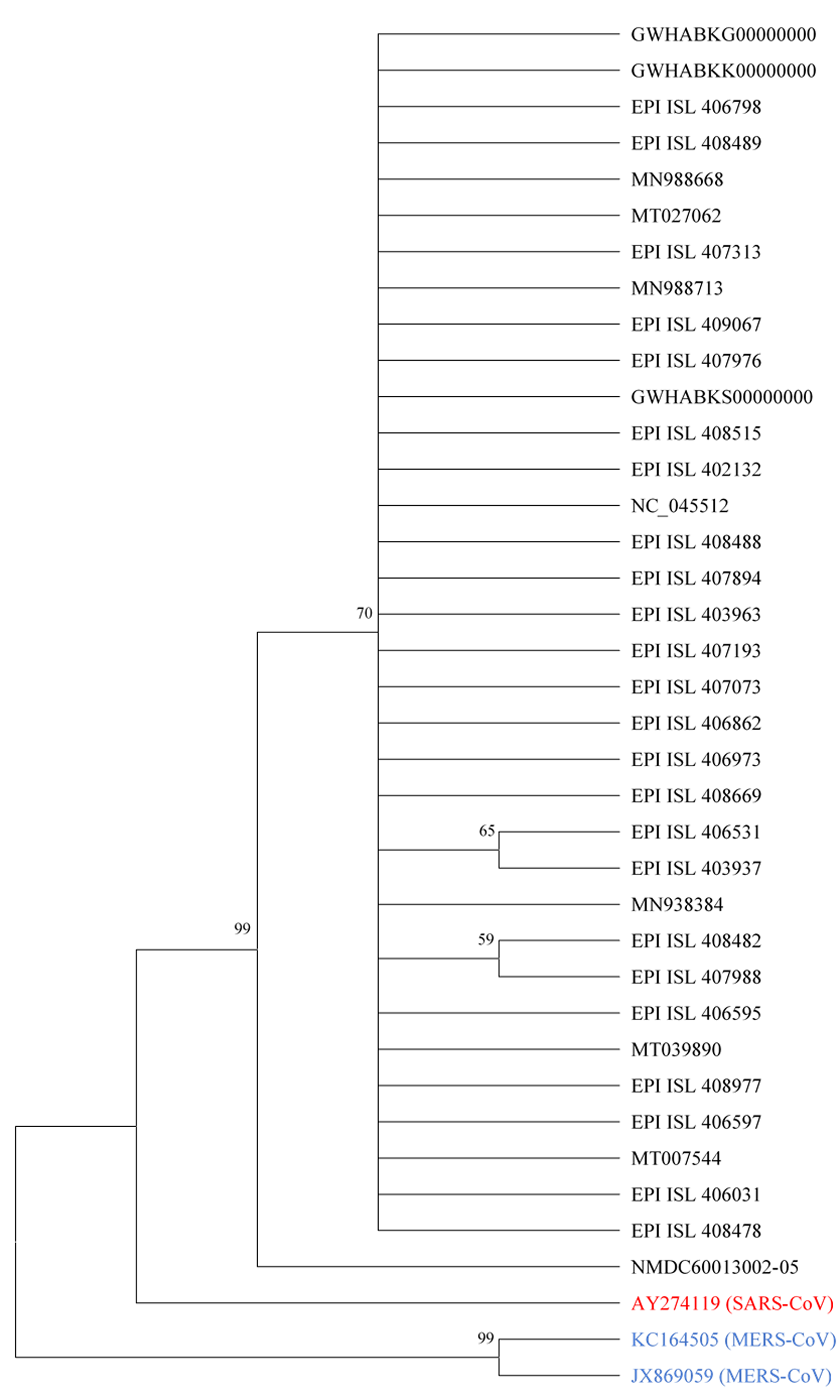

Supplement: Supplementary file 1 [file viruses-12-00244-s001.zip › Figure S1.tif]
